# Tell Me Where You Live… How the Perceived Entitativity of Neighborhoods Determines the Formation of Impressions About Their Residents

**DOI:** 10.3389/fpsyg.2022.821786

**Published:** 2022-03-15

**Authors:** Fátima Bernardo, José Manuel Palma-Oliveira

**Affiliations:** ^1^Department of Psychology, University of Évora, Évora, Portugal; ^2^CITUA - Center for Innovation in Territory, Urbanism, and Architecture, IST, Universidade de Lisboa, Lisbon, Portugal; ^3^CICPSI, Research Center for Psychological Science, Faculdade de Psicologia, Universidade de Lisboa, Lisbon, Portugal

**Keywords:** entitativity, impression formation, neighborhood, intergroup relationships, stereotypes

## Abstract

The studies presented here apply the concept of entitativity in order to understand how belonging to a particular geographical area – neighborhood - can determine the way others organize information and form impressions about area’s residents. In order to achieve this objective, three studies were carried out. The first study aims to verify if a neighborhood varies in terms of perceived entitativity, and identify the physical and social characteristics of the neighborhoods that are more strongly associated with the perception of entitativity. The Study 2 and 3 used an experimental paradigm to explore how people’s perceptions of neighborhoods’ entitativity influenced their impressions of residents. To activate stereotypes, Study 2 used the name of real neighborhoods, and Study 3 employed only a set of pictures of unknown neighborhoods. The results show that the neighborhoods vary significantly with the regard to the perception of entitativity, and a set of physical attributes of place were strongly related with entitativity. The results showed that, independent of stimuli, the neighborhoods perceived as highly entitative, the supposed residents were subject to more extreme and quicker trait judgments, supported by greater confidence on the part of perceivers. Study 3 also reported that in highly entitative neighborhoods, the perceivers transferred more traits from the group to individual members. These results provide strong evidence that physical structure of neighborhoods imply different entitatity judgments that influences the way in which residents are perceived.

## Introduction

The aim of this manuscript was to understand if the place of residence, specifically the neighborhood, can be an important source of information for forming impressions about its residents. As we know from social perception, our first impressions of others (individuals and groups) begin with visible cues that include physical appearance, non-verbal communication, behavior and the environment, namely living spaces (e.g., Carlston, 1994; Gosling et al., 2008). Using the available information the perceiver develops a mental conception of an individual or a group and uses that information to make judgments about people and groups. Although numerous studies focus on the cues used in forming impressions, the environments that people construct and inhabit have been little studied as a source of information for forming impressions about individuals and groups. One exception is the work of Gosling et al. (2002), which studied two personal environments: bedroom and office. They asked observers to rate their impressions about the occupant of an office or a bedroom, based only on the physical features of the environment. The results showed that the observer’s evaluation was quite accurate in comparison with evaluation by friends of the room or office occupant and the occupant’s rating of himself. This research provided strong support for the importance of personal environment in forming impressions about subjects. More recent publications have supported these findings in different contexts. In a domestic situation, Perez-Lopez et al. (2017) showed that observing primary spaces allowed researchers to make inferences about residents’ socio-demographic characteristics and Big Five personality traits In a second study two types of clues were identified that facilitated researcher inferences about residents: functional and symbolic ones which related to personality traits and sociodemographic characteristics. Poggio et al. (2013) had previously shown that competence and warmth, as set out in the Stereotype Content Model, were associated with the functional and symbolic categories of the objects, respectively. Results differed in the work context, where workspace personalization reflected more closely organizational policy rather than worker personality (Wells and Thelen, 2002; Wells et al., 2007).

The research presented in this chapter refers specially to the importance of neighborhood of residence as a source of information for forming impressions about its residents. Since the studies by Kevin Lynch, the theme of neighborhoods has acquired particular relevance in environmental cognition. Lynch (1976) identified neighborhoods (districts) as one of the five most probable categories of indicators used by people for structuring information about cities. In connection with Lynch’s work and in parallel with the emergence of social cognition, Terence Lee (Bartlett’s student) used Bartlett’s concept of schema to understand how people elaborated information about places. Specifically, Lee (1968, 1976) argues that the representation of space followed the same dynamic principles observed in the operation of social schemata and object schemata. The author used the term socio-spatial schema, as a particular type of schema used in space representation. He argued there was an isomorphism between the built environment and the social system where it was integrated (Lee, 2003). In this sense, it was impossible to isolate the physical environment from social meanings and behavioral activity patterns. “It is as if their very close interdependence has built up a mental organization coalescing them into a single functioning unit serving as a model for behavior” (Lee, 1962, cited in Lee, 2003).

The present research was focused on the study of “neighborhoods.” These are usually described as social categories (e.g., Spencer-Rodgers et al., 2007). Here we understand social categories in the sense of Social Identity Theory (Tajfel and Turner, 1979). Therefore, in the sense people define themselves as belonging to a neighborhood and have the perception that they share some characteristics with the other residents, this neighborhood can be understood as a social category that replicates processes of salience, discrimination, and optimal distinctiveness similar to those elicited by the more recently defined (non-spatial) social categories (Bernardo and Palma-Oliveira, 2016a; see Bernardo and Palma-Oliveira, 2016b). By showcasing ingroup/outgroup social identity processes involved in neighborhood belonging, this line of research illustrated self-stereotyping (self-categorization) *and*, necessarily, normal outgroup stereotyping. This is to say that neighborhood residents can be understood by others as sharing significant attributes, and in this sense they are perceived as a group. Thus, spatial/urban groups can be assumed to have the same proprieties as groups defined merely by social categories. Likewise, they differ not only as members of the ingroup perceived their relevance and centrality for their social identity, but also as they are evaluated by non-members. One of the most important factors that can sustain evaluations is that of perceived entitativity.

In this sense, the neighborhood was understood as a molar unit and an entity. However, as pointed out by Galster (2001), some neighborhoods are more perceived as a unit and more easily identified and geographically defined than others. The first objective of the present manuscript is to verify if neighborhoods varies in terms of perceived entitativity, and identify the physical and social neighborhood features that are related with perceived entitativity (Study 1).

The second objective is to explore the impact of residence in a more or less entitative neighborhood on the way others organize information and form impressions of hypothetical residents. Two sources of information were used to activate or develop the stereotype, the name of real neighborhoods (Study 2) and photographs of unknown neighborhoods (Study 3).

### Social Perception and Entitativity

To understand the degree of group unity, Hamilton and Sherman (1996) reintroduced Campbell’s (1958) concept of “entitativity,” which “refers to the degree to which a social aggregate is perceived as “having the nature of an entity, or having real existence” (p. 17). For Campbell, entitativity is not a group property that is present or absent in a group, but groups vary in the extent to which the perceivers see them as possessing this quality (i.e., entitativity). For instance, comparing a group of Gypsies and the high school basketball team, the first group is perceived to have much greater levels of entitativity than the second. Two aspects must be taken into consideration: firstly, any factors potentially associated with the perception of groups’ entitativity (entitativity antecedents); and secondly, the consequences in terms of information processing and impression formation regarding membership in groups with high or low entitativity.

### Entitativity Antecedents

A literature review, based on Hamilton et al. (2002) regarding the variety of cues on which entitativity might be based, allowed identification of a wide range of group properties. Following Campbell’s (1958) suggestion, a set of authors focused on the perceived similarity or variability among group members as a cue to perceived entitativity (e.g., Brewer et al., 1995; McGarty et al., 1995; Brewer and Harasty, 1996; Yzerbyt et al., 1998). Some studies found significant correlations between entitativity and similarity measures (e.g., Lickel et al., 2000; Spencer-Rodgers et al., 2007).

Another view suggested a connection between perceived entitativity and perception of interdependence, interconnectedness and organization among groups (e.g., Gaertner and Schopler, 1998; Hamilton et al., 1998; Lickel et al., 2000). Lickel et al. (2000) evaluated the interdependence among group members using three variables: interaction, common goals and common outcomes. They also used as a variable the importance of group membership in relation to the person’s interdependence on the other group members. In three studies, they verified that the group properties most strongly related to entitativity were interaction, common goals, common outcomes, group-member similarity, and the importance of the group. On the contrary, group size, length of group history and the permeability of group boundaries were not correlated with entitativity.

### Entitativity Consequences: Perceived Entitativity and the Perception of Group Members

The information processing about members of groups perceived to be highly entitative, i.e., with more unity and coherence, will be similar to the process used in forming impressions about individuals. These processes are summarized by Hamilton et al. (1999), as *integrative processing*.

In a set of studies, McConnell et al. (1994, 1997) verified that the entitativity perception of a target (individual or group) determines the mechanism of information processing invoked. Perceptions of high entitativity lead to an integrative impression of the target, resulting in an on-line judgment. Perceptions of low entitativity result in a memory-based judgment. More recent results have shown that information about high and low entitativity groups are represented in memory in differing manners (Johnson and Queller, 2003). Judgments about low entitativity groups involve verification of specific behavioral exemplars. On the contrary, judgments about high entitativity groups involve abstracted trait knowledge.

In studies more closely aligned to our own, research has identified judgment polarization in highly entitative groups. Dasgupta et al. (1999, Experiment 2) observed more negative impressions of highly entitative groups. On the contrary, Susskind et al. (1999) compared impressions of individual and group targets using only desirable attributes and found more favorable ratings in high entitativity targets. Both studies revealed that participants gave more extreme (positive or negative) ratings to groups with higher perceived levels of entitativity. The same results were found by Thakkar (2001).

Another recent finding demonstrated that groups perceived to be highly entitative are the only ones to reveal in-group extremity, both severe evaluation of negative in-group targets and more favorable evaluation of positive in-group targets (Lewis and Sherman, 2010).

Several investigations have concentrated on the impact of perceived entitativity on individual group members as opposed to the perception of groups. They studied whether group entitativity has an impact on the inferences made by perceivers. Brewer and Harasty (1996) stated that perceived entitativity might mediate the relationship between the stereotype of a group and impressions of group members. The perceived interchangeability in highly entitative groups, i.e., the perceptions of group members as sharing the same traits, facilitates stereotyping. Yzerbyt et al. (1998) explored the relationship between entitativity and social attribution, using Ross’s fundamental attribution error (Ross et al., 1977), and showed that membership of a highly entitative group influenced the explanation of individual members’ behavior, i.e., the use of fundamental attribution error only occurred for members of the more entitative groups. Rogier and Yzerbyt (1999) reported similar results.

Research has also shown that in more highly entitative groups, members are submitted to higher intra-group comparison than members of low entitative groups (Pickett, 2001) whilst the perception of high entitativity groups results in faster comparison than that of low entitativity groups (Pickett and Perrott, 2004).

However, few studies have investigated the direct impact of entitativity on stereotyping of existing groups. One was carried out by Crawford et al. (2002). They investigated the impact of perceived group entitativity on the processing of information regarding individual group members as well as the possibility that this information be transferred to another group member. They brought together Hamilton and Sherman’s position (1996) that an on-line abstraction of stereotypes occurs in high entitativity groups as well as Brewer and Harasty’s (1996) proposal that perceivers are more likely to have a prototypical representation of high entitativity groups but have an exemplary representation of low-entitativity groups. They verified their hypothesis that members of high entitativity groups are treated as interchangeable parts, and the transference of traits from one member to another is stronger than in low entitativity situations. To summarize, this review of entitativity research leads to the conclusion that the perception of group entitativity affects both the processing of information and the outcomes of this processing.

### Entitativity and Places

This project studied “neighborhoods” loosely described as social categories (e.g., Spencer-Rodgers et al., 2007). Neither this approach nor the application of the concept of entitativity are completely new, since Abrams (2006) considered the neighborhood as a meaningful entity. However, he studied the impact of neighborhood entitativity on residents’ identity, rather than the impact of neighborhood entitativity on neighborhood or residents’ perception.

The centrality of resident identity in most research into place and neighborhood identity show a lack of understanding that a certain local identity can be only understood as opposed/compared/evaluated to another neighborhood. The systematic parallel between places/neighborhoods and social categories that can both show the same kind of in-group/outgroup phenomena predicted by social identity, self-categorization and social cognition theories has been rarely carried out (e.g., Bonaiuto et al., 1996; Valera and Guardia, 2002 and essentially Bernardo and Palma-Oliveira, 2016b).

More recently, Lewicka et al. (2019) used photographs to confirm whether the concept of essentialism can be applied to places. Moreover, they replicated the double nature of the concept previously described in relation to social groups by authors such as Haslam et al. (2004) and Demoulin et al. (2006). This double nature included, on the one hand, entitativity, associated with perceptions of homogeneity, stability and enclosure of places and, on the other, place specific essentialism associated with historicity, naturalness, distinctiveness, sense of insidedness, and the genius loci of a place. Although not its primary aim, this study highlighted the relevance of entitativity for understanding spaces, as stated: “The concept of entitativity may be a more appropriate way to understand the perception of artifacts, such as places, than essentialism is” (Lewicka et al., 2019, p. 4).

In line with work on space essentialism Lewicka et al. (2019) and Wnuk et al. (2021), compare the degree of place essentialism with the way the places were understood as a place that allows different degrees of ethnic diversity. The results support the hypotheses that essentialist places (more associated to bounded entity with historical continuity) were related with less openness toward the presence of ethnic out-group members than anti-essentialist locations (that are more open to change). These results support the importance of understanding the impact of place features on the perception of places and heir residents.

## Study 1

This study looked at the antecedents of perceived entitativity in group perception based on a geographic dimension, i.e., neighborhoods. The underlying premise of this study was that urban aggregates, such as neighborhoods, vary in terms of perceived entitativity. Three specific issues regarding the perception of entitativity in neighborhoods were analyzed. Firstly, if an urban aggregate such as a neighborhood could be perceived with a certain degree of entitativity. Secondly, if it was possible to identify the social and physical characteristics of these neighborhoods that were more strongly associated with the perception of entitativity. And finally, if it was possible to identify different dimensions in the perception of neighborhoods.

### Method

#### Pre-test

The pre-test was designed to choose a group of neighborhoods in the city of Lisbon that were better known by students, both in terms of geographical location and characterization. A questionnaire was designed in order to assess knowledge about 47 neighborhoods all over the city, using three questions for each neighborhood: (1) “Have you ever heard of this neighborhood?”; (2) “Can you identify where it is situated?”; (3) “Have you ever been to this neighborhood?.” Following their informed consent, 50 Technical University of Lisbon civil engineering students and Higher Institute of Applied Psychology, psychology undergraduates answered a questionnaire. The 20 districts included in the main study were based on the following criteria: 100% of subjects responded positively to question 1; at least 70% of participants responded correctly to question 2; and at least 50% responded yes to question 3.

### Main Study

#### Participants

A total of 189 university students from the Technical Institute and the Higher Institute of Applied Psychology, both in Lisbon, participated in this study. The sample consisted mainly of residents that live in the metropolitan area of Lisbon (MAL) permanently (73.5%), and only 26.5% lived in MAL during the school year, returning home in the summer. The sample was composed of 42.3% female and 57.7% male students, with an overall mean age of 22.24 years (*SD* = 2.632). The questionnaire was answered in a classroom context.

#### Material

The twenty two properties included: (a) three questions about neighborhood preferences (like/dislike; positive/negative and good/bad; (b) thirteen questions about the physical characteristics of neighborhoods (e.g., size, degrees of organization, attractiveness, and modernity). These questions were selected from the environmental adjectives by Kasmar (1970), as the most relevant to the study objective and context. (c) six questions about group properties, which included entitativity, group member similarity, interaction, importance, goals, and outcomes. These group properties, in a previous study, revealed a strong positive correlation with entitativity (Lickel et al., 2000, Studies 1 and 2). To evaluate entitativity, a scale ranging from 1 (not a group at all) to 9 (very much a group) was used as per Lickel et al. (2000).

The last section of the questionnaire was socio-demographic characterization, which included questions about sex, age, birthplace, place of residence, and the frequency of visiting each evaluated neighborhood.

#### Procedure

Since the questionnaire would have required participants to rate each neighborhood in twenty-three characteristics, evaluating 20 neighborhoods was assumed to be onerous. The task was, therefore, divided into four application conditions (a procedure used for example in Slovic et al., 1985). All participants rated only eight neighborhoods, with four neighborhoods being the same for all participants (Parque das Nações, Chelas, Lapa, and Bairro Alto) and the other four different for each condition. This procedure allows us to understand statistically *a posteriori* whether we can use the questionnaires from the different groups as if they were from the same population.

Subject distribution over the four conditions was randomized. 50 participants responded to the first condition (26.5%), 50 to the second (26.5%); 44 to the third (23.2%) and 45 to the fourth (23.8%). To control for possible order effects, the neighborhoods in the four conditions appeared at random.

In order to analyze the data from the four conditions as if they were part of the same sample, some demographic characteristics of the four conditions were statistically compared. No statistical differences were found between the four experimental conditions in relation to gender X2 (3) = 4.679, *p* < 0.197, age *F* = 0.373, *p* < 0.773 or place of residence *F* = 1.609, *p* < 0.189.

Participants were asked to rate some Lisbon neighborhoods according to 22 properties. The neighborhood name appeared in capital letters at the top of the page, together with instructions. Subjects were asked to focus on the characteristics of each area before rating each neighborhood on a nine-point scale.

Upon completion of the questionnaire, participants were thanked for their participation and provided with an email in case they wanted additional information and/or to receive the study results.

### Results

#### Perceived Entitativity

The underlying premise of this study was that urban aggregates, such as neighborhoods, vary in terms of entitativity. Participants’ average ratings for each neighborhood are presented in Table 1. The results show an important variation between the groups, i.e., participants perceived the neighborhoods with a substantial variation in terms of entitativity, with a mean group entitativity ranging from 7.36 to 3.41.

**TABLE 1:** Perceived entitativity rating for 20 neighborhoods (mean and SD).

Neighborhoods	Mean	*SD*
Alfama	7.36	1.57
Castelo	6.86	1.47
Graça	6.47	1.78
Chelas	6.26	2.22
Bairro Alto	6.19	1.71
C. Ourique	5.86	1.44
Pontinha	5.80	1.41
Encarnação	5.74	1.79
Belém	5.60	1.53
Ajuda	5.51	2.05
Lapa	5.47	1.93
Alvalade	5.26	1.87
Baixa	5.18	1.88
Intendente	5.14	2.14
Olivais	5.06	2.06
Anjos	4.83	1.89
Benfica	4.70	1.93
Restelo	4.67	2.08
Telheiras	4.66	1.82
P. Nações	3.41	1.99

#### Entitativity and Neighborhoods’ Social and Physical Properties

The second aim of this study was to identify the social and physical characteristics of those neighborhoods perceived to be greater in entitativity. A variable correlation matrix was formed, as shown in Table 2. The results showed a strong and positive Pearson inter-correlation between all the group properties assessed. Perceived interaction was the variable most highly correlated with entitativity (*r* = 0.96), and the similarity property was the least correlated (*r* = 0.73).

**TABLE 2:** Correlation among group properties rating.

	Entitativity	Interaction	Importance	Goals	Outcomes	Similarity
Entitativity	___	0.96**	0.74**	0.83**	0.77**	0.73**
Interaction		___	0.62**	0.75**	0.71**	0.63**
Importance			___	0.77**	0.67**	0.74**
Goals				___	0.96**	0.92**
Outcomes					___	0.91**
Similarity						___

**Correlation is significant at the 0.01 level.

Concerning physical properties, the Pearson correlation between neighborhoods’ physical characteristics and group properties, in particular the perceived entitativity, is presented in Table 3. The results showed a strong negative correlation between entitativity and the following neighborhood properties: modern, functional, organized, well-planned, rich, and large. Thus, the physical properties that participants associated with the entitativity of a neighborhood were a small and poor area, old, not necessarily functional, and not necessarily well-organized or planned, which in the general public’s view represent the more traditional neighborhoods in central Lisbon.

**TABLE 3:** Correlation among group properties and physical properties.

	Entitativity		Interaction		Importance		Goals		Outcomes		Similarity	
Attractive	–0.05		–0.22		0.58	**	0.16		0.06		0.19	
Clean	–0.4		–0.57	**	0.17		–0.13		–0.22		–0.06	
Modern	–0.78	**	–0.80	**	–0.61	**	–0.55	*	–0.48	*	–0.42	
Unique	0.36		0.25		0.83	**	0.46	*	0.34		0.45	*
Functional	–0.71	**	–0.80	**	–0.25		–0.47	*	–0.49	*	–0.43	
Organized	–0.65	**	–0.76	**	–0.13		–0.39		–0.45	*	–0.34	
Inviting	0.2		0.04		0.73	**	0.31		0.19		0.30	
Well-balanced	–0.24		–0.41		0.35		–0.05		–0.15		–0.03	
Well-planned	–0.69	**	–0.75	**	–0.27		–0.46	*	–51	*	–0.44	
Good	–0.11		–0.28		0.53	*	0.09		0		0.11	
Consonant	–0.28		–0.43		0.31		–0.09		–0.20		–0.06	
Rich	–0.51	*	–0.67	**	0.12		–0.22		–0.28		–0.16	
Large	–0.65	**	–0.75	**	–0.42		–0.44		–0.43		–0.30	

*Significantly different at p < 0.05, **significantly different at p < 0.01.

#### Dimensions in Neighborhood Perception

The last goal of this study was to explore the possibility of identifying different dimensions in the perception of neighborhoods, i.e., if it was possible to aggregate the properties in main factors, to understand the neighborhoods’ perception. To achieve this purpose, a Principal Components Analysis (PCA) was carried out. For this we averaged the ratings for each of the 22 attributes over participants and aggregated the data across neighborhoods (following the procedure described by Maroco, 2021) and performed a PCA on this data with a varimax rotation. Three items were excluded (“pleasant,” “positive,” “I like”), because correlations with other items were greater than 90%.

As seen in Table 4, three factors were extracted with 65.59% of variance explained. The first factor was denominated “attractiveness” and explained 24.808% of variation. The second factor, “functionality,” justified 21.242% of the variation and was negatively related to entitativity. The third factor, “group connectiveness,” explained 19.536% of variation, and included all the social features considered. The Cronbach Alfa for the group of dimensions associated with each factor was 0.856, 0.859, and 0.870.

**TABLE 4:** PCA loading with a varimax rotation.

	“Attractiveness”	“Functionality”	“Group connectiveness”	Communalities
Good	**0.861**	0.275	–0.004	0.817
Attractive	**0.849**	0.170	–0.023	0.750
Inviting	**0.837**	0.046	0.116	0.716
Well balanced	**0.722**	0.392	0.038	0.676
Unique	**0.629**	–0.230	0.197	0.488
Consonant	**0.626**	0.442	0.034	0.589
Rich	**0.621**	0.507	–0.163	0.670
Modern	–0.069	**0.824**	–0.175	0.714
Organized	0.433	**0.721**	–0.074	0.712
Functional	0.368	**0.708**	–0.047	0.639
Well planned	0.336	**0.683**	–0.047	0.581
Large	–0.020	**0.671**	–0.060	0.454
Clean	0.545	**0.598**	–0.118	0.668
Common goals	0.024	0.029	**0.878**	0.772
Common outcomes	–0.066	0.083	**0.830**	0.700
Similarity	0.003	0.044	**0.815**	0.666
Entitativity	0.025	–0.309	**0.739**	0.642
Interaction	–0.027	–0.404	**0.719**	0.680
Importance	0.310	–0.143	**0.631**	0.526
*Alfa Cronbach*	0.856	0.859	0.870	
*Eigenvalues*	6.630	4.034	1.797	
*Variance*	24.808	21.242	19.536	

*The bold highlights the items that contribute to each factor.*

### Discussion

The results showed clear evidence of wide differences in the entitativity perception of different neighborhoods by residents of the city. Thus, participants perceived the neighborhoods on a continuum from high entitativity neighborhoods (Alfama) to low entitativity neighborhoods (Parque das Nações). Therefore, perceived entitativity appears to be as easily applied to neighborhoods as the other social perception processes usually attributed only to classic social groups.

The results also identify the social and physical characteristics of the neighborhood most strongly associated with the perception of entitativity. Concerning social characteristics, the results showed a strong positive relation between entitativity and the following perceived properties: interaction, common goals, common outcomes, group member similarity and importance of the group. These results were similar to those obtained by Lickel et al. (2000, Studies 1 and 2) for social groups.

This research also made a connection between perceived entitativity and physical characteristics of the space, identifying a set of physical properties of neighborhoods that were strongly correlated with entitativity, namely “traditional,” “not functional,” “not organized,” “not well-planned,” “small,” “poor,” and “unique.”

These results revealed that groups defined by belonging to a neighborhood could generate the same type of processes that were well identified in the perception of individuals and groups (Hamilton and Sherman, 1996). People were found to make inferences between the physical and social characteristics of the neighborhood. These inferences were consistent among individuals and neighborhoods. So we could suppose that people had consistent “implicit theories” about the city, as in relation to other types of groups and individuals, which integrate both social and physical characteristics of the neighborhoods.

It is important to realize the relevance of these results in designing and planning new areas of the city or in the renovation and revitalization of older areas. In this connection, two important questions may have to be answered. The first is what is the importance of designing neighborhoods with high entitativity? Several studies show that people identify more strongly with high entitativity groups (e.g., Yzerbyt et al., 2000; Castano et al., 2002, 2003; Hogg et al., 2007). Also, besides the well-known importance of place identification for people, much research shows that identification with the place of residence increases the feeling of security (e.g., Brown et al., 2003; Félonneau, 2004), increases the maintenance of place (e.g., Stedman, 2002; Brown et al., 2003), promotes pro-environmental behavior (e.g., Pol et al., 2002; Uzzell et al., 2002), ecological behaviour (e.g., Vaske and Kobrin, 2001; Vorkinn and Riese, 2001; Bonaiuto et al., 2008), community involvement and public participation. Therefore, one important objective of urban planners is to design places that facilitate appropriation and identification (e.g., Lynch, 1976; Norberg-Schultz, 1980). Bernardo and Palma-Oliveira (2016a,b) have also shown the importance of positive social and place identity.

The second question is how the results of the present study can contribute to designing higher entitativity neighborhoods. In this respect, this study identifies two main dimensions of neighborhoods’ physical properties: attractiveness and functionality. It showed that entitativity was negatively correlated with functionality and in relation to attractiveness it is only positively correlated with one property of the attractiveness dimension, namely, “unique.” Also, that entitativity is strongly correlated with social dimensions such as interaction. So in order to promote the development of more entitative neighborhoods it is important to build areas that promote and facilitate social interaction, as well as make spaces that are unique, in the sense that is easy to distinguish them from the spaces round about.

In this study, the size of the neighborhood was strongly associated with its entitativity, that is, small neighborhoods were perceived as more entitative than larger neighborhoods. These results are supported by Brewer et al. (1995) and Brewer and Harasty (1996) and consistent with the Mullen (1991) model of category representation. This model predict that the cognitive representation of minority groups members will be category-based, and more perceived as entities, in comparison with the majority group members (Brewer et al., 1995, Study 3). Previous research showed that group size affects the quality and quantity of social interaction (e.g., Cohen and Cohen, 1991, cited in Lickel et al., 2000). Accordingly, Lickel et al. (2000) showed that group size may not be directly linked to entitativity, but scale has an indirect effect insofar as it affects the interaction of group members. And interaction was the dimension most strongly associated with entitativity.

## Study 2

Studies 2 and 3 applied the social psychology notion of entitativity (e.g., Hamilton et al., 2002) to understanding how people organize information and form impressions about places and their residents. Groups perceived to be highly entitative were expected to differ from those perceived to have low entitativity and with an integrative impression of the target expected (e.g., Hamilton et al., 1999; Thakkar, 2001; Johnson and Queller, 2003), resulting in on-line judgment. More extreme trait judgment and faster responses were predicted, as was greater confidence in the judgment than for groups perceived to be less entitative. In low entitativity groups, memory-based judgments were expected to be invoked, resulting in less extreme trait evaluation, lower responses, and lesser judgment confidence.

### Predictions

This study compared how people organize information and form impressions about two neighborhoods that differed in terms of perceived entitativity: one with high entitativity and other with low entitativity. Thus, it was predicted that participants judging high entitativity neighborhoods would: (H1) make more extreme trait ratings, (H2) respond faster and (H3) have more confidence in their judgments than participants forming opinions about low entitativity groups.

### Method

#### Overview

The procedures used in this study follow closely the paradigm described by Susskind et al. (1999). Participants were informed that the study objective was to examine how people form first impressions. After that, they were exposed to a set of sixteen statements describing behaviors of members of a group. The name of the neighborhood was identified after the descriptive statements. Each participant only answered in relation to one group. After a filler task, the participants rated the thematic traits and their confidence in making judgment. Finally, participants completed a questionnaire about their perception of group entitativity.

The pre-test was designed to select the groups to use. Two neighborhoods, one perceived to have low entitativity and another perceived as highly entitative were selected based on Study 1.

#### Main Study: Participants and Design

An experimental study was conducted with 82 psychology students from the University of Lisbon, who received course credits for their participation. The subjects were randomly assigned to two target conditions. Thus the experiment consists of a 2 (entitativity: high vs. low).

The sample consists of students who are permanent residents of the Metropolitan Area of Lisbon and was composed of 77% female and 13% male students, with an overall mean age of 21.78 years (*SD* = 4.27).

#### Material

All the materials were presented using E-Prime software, in a laboratory, in separate cubicles, in order to record the answers and the latency time. The statements presented to participants describe behaviors of target group members. Each sentence had a different male name at the beginning. The behaviors were selected to give information about four themes: athleticism, sociability, political activism, and intelligence (based on Susskind et al., 1999).

#### Procedure

##### Initial Instructions

The experiment was carried out in the laboratory and all the instructions were provided by computer. On the first screen the participants read that the main objective of the study was to understand how people form first impressions. After that they read the following instructions:

*‘A set of statements are presented describing behaviors of Alfama residents.*

*Each behavior has been performed by a different resident.*

*Thus, we ask you to form an impression of the residents of this place.’*

The instructions the neighborhood name in bold letters.

Sixteen statements describing group member behaviors were presented (e.g., for sociability, “Listened to a friend who wanted to discuss a problem”; for political activism, “Listened to the candidates debate on the radio”; for intelligence, “Worked on a difficult mind puzzle for enjoyment”; for athleticism, “Enjoyed learning to play a new and difficult sport.” The 16 sentences from the four themes were displayed individually on the computer screen, each one for 6 s and in random order. The participants then completed a 3-min filler-task consisting of counting the number of times the letter “E” appeared in a text. Next, they completed four dependent measure tasks: a trait judgment task, confidence judgment, and a perceived entitativity measure.

#### Dependent Measures

##### Trait Judgment Task and Confidence Judgment

Participants were asked four judgment questions in relation to the four sentence themes (based on Susskind et al., 1999) (e.g., “How intelligent do you think the residents of this neighborhood are?”). Each question appeared on the computer screen and the participant was asked to type the number corresponding to their response, on a seven-point Likert scale. Because the response latencies were recorded (in milliseconds), this task was preceded by a group of four questions to familiarize the participant with the procedure. Participants were also asked about their level of confidence in their judgment regarding the four themes on a seven-point Likert scale (e.g., “How confident are you in how intelligent the residents of this neighborhood are?” Both the rating scores and the response latencies were recorded.

*Perceived entitativity measures –* the perception of entitativity was assessed with eight items adapted from Spencer-Rodgers et al. (2007, Study 2) (e.g., “Some groups have the characteristics of a ‘group’ than others do. To what extent does this group qualify as a group?”). The items were rated on a nine-point scale ranging from 1 (not at all) to 9 (extremely). The Cronbach’s Alfa from the original scale for social categories was 0.86 and in the present study was 0.93. For the neighborhood groups, participants were also asked to rate them on a nine-point scale concerning the following physical characteristics: “modern,” “functional,” “organized,” “well-planned,” “large,” “rich,” and “not unique.” In Study 1, these physical characteristics showed a strongly negative correlation with entitativity.

### Results

#### Check of Physical Differences Between Neighborhoods

To verify if participants knew the physical characteristics of the neighborhoods, answers regarding these were submitted to a *t*-test. The results (Table 5) showed significant differences between both neighborhoods. The results also confirmed the previous premise that neighborhoods perceived with high entitativity would have lower scores on the physical characteristics studied than less entitative neighborhoods.

**TABLE 5:** Perceived physical characteristics of neighborhoods - means rating and *t*-student.

	High entitativity neighborhood (Alfama)	Low entitativity neighborhood (P.N.)	*t*	*p*
Modern	3.12	7.49	–11.351	0.000
Functional	5.05	6.61	–4.496	0.000
Organized	5.12	6.37	–3.798	0.000
Well Planned	4.78	6.93	–5.352	0.000
Rich	4.17	7.68	–12.078	0.000
Large	3.76	6.98	–9.650	0.000

#### Check of Perceived Entitativity of Neighborhoods

The scores of the entitativity scale were submitted to an independent sample *t*-test, to confirm that the two neighborhoods are perceived with different degree of entitativity. The results showed significant differences between the two groups in relation to the entitativity scale *t*(1,80) = 5.327, *p* < 0.001, and in response latencies to the same scale *t*(1,80) = −4.218, *p <* 0.001. Participants on the high entitativity condition reported a higher entitativity (*M* = 5.93) than the participants on the low entitativity condition (*M* = 4.55). Concerning the response latencies to entitativity scale, participants on the high entitativity condition reported a low response latencies (*M* = 5071) than the participants on the low entitativity condition (*M* = 6519). This result confirm that neighborhoods are perceived as having different degrees of entitativity.

#### Trait Judgments and Confidence

The initial prediction (H1) was that, in relation to high entitativity groups, in our case neighborhoods, participants would make more extreme trait judgments than regarding low entitativity groups. Trait judgment was analyzed in a 2 (entitativity: high vs. low). An independent samples *t*-test with 41 participants per group (*N* = 82) would be sensitive to effects of Cohen’s *d* = 0.73 with 95% power (alpha = 0.05, two-tailed). This means the study would not be able to reliably detect effects smaller than Cohen’s *d* = 0.73.” The results partially supported the prediction, the participants in the high entitativity neighborhoods made significantly more extreme ratings than did those in the low entitativity neighborhoods for two of the four themes (sociability and political activism, see Table 6).

**TABLE 6:** Neighborhood judgment and confidence - mean rating and *t*-student.

	High entitativity neighborhood (Alfama)	Low entitativity neighborhood (P.N.)	*t*	*p*
**Trait judgments**				
Intelligence	4.71	4.68	0.133	0.895
Sociability	5.59	4.61	3.957	0.000
Athleticism	5.07	4.68	1.688	0.095
Political Act.	5.32	4.41	3.968	0.000
**Response latencies to trait judgment**				
Intelligence	4872	6267	–3.541	0.001
Sociability	4281	5949	–3.423	0.001
Athleticism	4956	6307	–2.234	0.028
Political Act.	4600	5782	–3.269	0.002

It was predicted that the level of perceived group entitativity would influence the way participants processed information about the group, more specifically, subjects were expected to make more on-line trait judgments about high rather than low entitativity groups, whether social categories or neighborhoods (H2). The results also support the prediction, (Table 6). Participants in high entitativity neighborhoods judged residents more quickly than those in low entitativity ones.

Finally it was predicted that perceptions of entitativity would influence confidence in judgment, i.e., the hypothesis 3 stated that participants judging high entitativity groups would view the behavioral information as more informative to make dispositional inferences in comparison to in low entitativity neighborhoods. Thus, it was expected that participants judging persons from high entitativity neighborhoods would report more confidence in their trait judgments than those evaluate persons from low entitativity places. Confidence was analyzed in a 2 (entitativity: high vs. low) × 4 (sociability, intelligence, political activism, athleticism Theme), ANOVA with repeated measures on the last variable.

The results do not support the prediction (Table 6), *F*(1,80) = 0.645, *p* < 0.424, no significant differences were found.

### Discussion

The goal of Study 2 was to investigate neighborhoods in a city and verify if the consequences of belonging to a specific neighborhood with high or low entitativity would have the same impact on impression formation as that identified for other types of groups (intimacy groups, task groups and social categories) (e.g., McConnell et al., 1994, 1997; Yzerbyt et al., 1998; Susskind et al., 1999). Particularly, we wanted to assess this with regard to how judgments were made (rating and response latencies), and the confidence in judgments. The results did not refute the hypotheses. Firstly, participants perceived the neighborhoods as different in terms of entitativity. Secondly, these differences in perceived entitativity had an impact on forming group impressions. Thus, participants made more extreme and faster judgments regarding neighborhoods perceived to be highly entitative than for low entitativity areas. These results were consistent with the idea that, in relation to groups perceived with high entitativity, people carry out a more integrative information processing, an on-line processing, than for groups perceived with low entitativity.

Information about a neighborhood’s residents can be organized either by way of a pre-existing stereotype of the neighborhood and its people, as was tested in Study 2, or may result from visual information about the neighborhood. In this case, will the physical characteristics of a neighborhood, by themselves, lead subjects to perceive a certain level of entitativity, which, in turn, might determine how people process information about its residents? In the Study 1 it was showed that neighborhoods that show high and low entitavitity shared a very specific set of physical characteristics. Thus our hypothesis was that those features would be acting as sufficient clue to stereotype activation. That was the question guiding Study 3. It used merely pictures of neighborhoods as a source of information, or descriptions based merely on their physical characteristics.

## Study 3

### Predictions

This study was two main objectives: first replicate Study 2 using a different stimulus to activate the group stereotype. We used a set of photograph or a description of an unknown neighborhood based on the characteristics strongly correlated with entitativity (Study 1), as a stimulus to activate the neighborhood stereotype. The second objective was to evaluate the effect of perceived group entitativity on the transference of group stereotype to individual members. As mentioned above, the literature reports that, in high entitativity groups, group members will be perceived more according to the group’s stereotype than members of groups with less perceived entitativity (e.g., Brewer and Harasty, 1996; Crawford et al., 2002; Spencer-Rodgers et al., 2007).

Thus, it was expected that participants judging high entitativity neighborhoods would: (H1) make more extreme trait ratings, and (H2) respond faster. The last hypothesis (H3) predicted that participants judging high entitativity groups would more easily transfer behavioral traits from the group stereotype to individual group members than those in the low entitativity condition.

### Method

#### Overview

In this third study, photographs and descriptions of the neighborhoods were used to induce development of the group stereotype. They were based on the physical characteristics strongly correlated with perception of entitativity (study1). The procedures followed closely the paradigm described by Susskind et al. (1999) and were the same as in Study 2.

A procedure inspired by Crawford et al. (2002, Studies 1 and 2) evaluated the transference of group stereotypes to individual group members. At the end, participants were informed that a resident of the neighborhood previously discussed would be described. A list of six adjectives was presented for 10 s. The participants then carried out a filler task lasting 3 min. The filler was to find a list of 20 city names in an array of letters. Afterward, a list of fourteen adjectives was presented in a random order to verify if participants attributed characteristics of the group to the individual group member.

#### Pre-test

The pre-test served to select the photographs to generate the neighborhood stereotype in the subsequent study. The test was to confirm if the photographs were identified with the physical properties referred to before and if they were perceived as characteristic of high or low entitativity groups. Two sets of photographs were collected: one that represented the high entitativity neighborhood and another that represented the low entitativity neighborhood. Based on the Study 1, the photographs for the high entitativity group needed to have the following characteristics: small, poor, traditional, poorly planned, non-functional and disorganized. Photographs for the low entitativity group needed to have the following characteristics: large, rich, modern, well-planned, functional and organized.

Two sets of eight pictures were chosen for the test, one set for each level of entitativity. Sixty students from the University of Évora (the same university where the main study would be carried out) rated both groups of photographs according to 6 physical characteristics (small/large; poor/rich traditional/modern; poorly planned/well planned; non-functional/functional; and disorganized/organized) as well as on the entitativity scale used in Study 1. There was also a question about possible identification of the city in the photographs, to control for the effect of previous stereotypes in relation to the city. All the respondents volunteered to participate and provided their informed consent. The pre-test was performed in the classroom context. The participants were exposed to the entire set of photographs for 6 s each on the screen. Then all participants rated the group of photographs according to the 6 physical properties, and the entitativity scale, using a nine-point scale.

To control for any possible effect of order, the two groups of photographs alternated. Half of the participants saw the high entitativity before the low entitativity then the order was reversed for the second group. In order to analyze the data from the two conditions as if they were part of the same sample, the ratings of the two conditions were statistically compared, and no statistical differences were found.

The main results of the questionnaire can be seen in Table 7, showing a significant difference between the two conditions for all variables. In relation to the entitativity scale, the Cronbach Alfa was 0.95. Nobody correctly identified the geographical location of the photographs.

**TABLE 7:** Main results of the pre-test.

	High entitativity condition		Low entitativity condition		*F*	Sig
Properties	Mean	*SD*	Mean	*SD*		
Small/large;	5.87	0.769	3.88	0.904	167.535	0.000
Poor/rich	6.42	0.619	3.75	0.836	394.360	0.000
Traditional/modern	6.38	0.121	2.37	0.780	467.692	0.000
Poorly planned/well planned	5.80	0.113	4.35	1.010	55.030	0.000
Non-functional/functional	5.88	0.101	4.12	0.976	94.948	0.000
Disorganized/organized	6.10	0.752	4.18	0.983	143.883	0.000
Entitativity Scale	4.04	0.728	5.93	0.546	258.564	0.000

### Main Study

#### Participants and Design

An experimental study was conducted with 121 psychology students at the University of Évora. All respondents volunteered to participate and provided their informed consent; they received course credits for their participation. The subjects were randomly divided into four target conditions. Thus the experiment consists of a 2 (entitativity: high vs. low) × 2 (type of group presentation: photographs vs. description).

The sample was composed of 84% female and 16% male students, with an overall mean age of 22.40 years (*SD* = 2.632).

#### Material and Procedure

All the materials were presented using E-Prime software, in a laboratory with separate cubicles. Participants also received a sheet to fill in their socio-demographic data and perform the filler tasks.

The experiment took place in two steps, the first one was similar to Study 2. The procedure included presentation of 16 statements describing behaviors by members of a group, followed by a filler-task, and two dependent measure tasks: a trait judgment task and response latencies to traits judgment.

The second part evaluated any transference of group stereotype to individual group members. The participants were informed that a resident of the previous presented neighborhood presented before named “Sebastião” would be described. A list of six adjectives appeared for 10 s (e.g., honest, persistent, attractive, and imaginative). Next, the participant completed a filler task that entailed finding a list of 20 city names in an array of letters. Subsequently, a list of fourteen adjectives was presented in random order as a recognition task. These adjectives included 6 synonyms of the adjectives presented before (with a similar frequency in the Portuguese language) (e.g., serious, insistent, fascinating, fanciful), the four adjectives of the four themes presented in the first part of the study (smart, friendly, and politically committed and athletic) and adjectives without any relation to the topics (happy, important, imaginative, and strong). For each adjective the participants had to answer whether they felt the adjective was characteristic of the resident described previously.

Finally the participants completed two measures: perceived entitativity measure and perceived physical characteristics of the neighborhood.

#### Dependent Measures

The dependent measures are similar to that used in Study 2.

The recognition task included the presentation of fourteen adjectives, one at a time, at random. Participants were instructed to evaluate whether or not each of the adjectives could be applied to Sebastião. The adjective list was put together to check whether participants perceived a correspondence between the list of Sebastião’s traits and the list of adjectives, or if they transferred some of the group characteristics to Sebastião. Two types of measures were used. *Trait inference* identified where participants pointed out the traits that were synonymous with the initial adjectives of the subject; *trait transference* indicated where participants would transfer traits of the group to Sebastião.

### Results

#### Check of Physical Differences Between Neighborhoods

To find out whether participants were aware of the physical characteristics of the neighborhoods, they evaluated the neighborhood in relation to the 6 physical characteristics at the end of the experiment. The ANOVA analysis indicated a main effect of the target type for both the photo condition and description condition. The Tukey HSD *post hoc* results are shown in Table 8.

**TABLE 8:** Means and *post hoc* tests – Tukey HSD – physical characteristics.

	Photographs			Description		
	High entitativity	Low entitativity	F Sig	High entitativity	Low entitativity	F Sig
Modern	2.29	6.30	0.000	4.86	7.07	0.000
Functional	4.16	6.03	0.000	4.10	6.66	0.000
Organized	4.55	5.67	0.062	4.93	6.34	0.012
Well planned	4.84	5.67	0.140	5.67	6.34	0.000
Rich	3.81	5.70	0.001	3.93	7.00	0.000
Large	3.97	6.37	0.000	4.10	6.24	0.000

#### Check of Perceived Entitativity

The scores of the entitativity scale were submitted to an ANOVA in a 2 (entitativity: high vs. low) × 2 (type of group presentation: photographs vs. description). A between-subject ANOVA with 121 participants across four groups would be sensitive to effects of η^2^ p = 0.18 with 95% power (alpha = 0.05). This meant the study would not be able to reliably detect effects smaller than η^2^ p = 0.18.

The results showed a significant main effect on the entitativity *F*(1,116) = 14.472, *p* < 0.002; η_*p*_^2^ = 0.111. Participants on the high entitativity condition reported a higher entitativity than the participants on the low entitativity condition for the photographs condition (*M* = 5.81, *M* = 4.65, p = 0.003), but not for the description condition (*M* = 5.52, *M* = 4.93, p = 0.268). Neither the main effect of type of groups [*F*(1,116) = 0.000, p = 0.992] nor the interaction between entitativity and type of groups achieved significance [*F*(1,116) = 1.450, p = 0.231].

The results confirmed the predictions and showed a significant main effect in response latencies in the entitativity scale, *F*(1,116) = 14.621, *p* < 0.001; η_*p*_^2^ = 0.112. Participants on the high entitativity condition reported lower response latencies than the participants on the low entitativity condition for the photographs condition (*M* = 5574, *M* = 6768, p = 0.038), but not for the description condition (*M* = 5655, *M* = 6862, p = 0.040). Neither the main effect of type of groups nor the interaction between entitativity and type of groups reached significance.

#### Trait Judgments

Four trait judgments were analyzed with MANOVA with 2 (entitativity: high vs. low) × 2 (type of group presentation: photographs vs. description) × 4 (sociability, intelligence, political activism, athleticism Theme), MANOVA with repeated measures on the last variable.

The results generally support the prediction, the main effect of the entitativity target was significant *F*(1,116) = 30.246, *p* < 0.001; η_*p*_^2^ = 0.207. Simple effects analysis shows that participants in high entitativity target report more extreme trait ratings, when we considered the sum of traits (see also Table 9).

**TABLE 9:** Means ratings – trait judgment and response latencies to trait judgment.

	Photographs			Description		
	High entitativity	Low entitativity	*p*	High entitativity	Low entitativity	*p*
**Trait judgments**						
Intelligence	4.42	4.83	0.878	4.60	4.79	0.998
Sociability	5.65	4.20	0.000	5.33	4.14	0.001
Athleticism	4.97	4.17	0.026	5.03	4.28	0.045
Political Act.	5.23	4.30	0.007	5.47	4.52	0.007
Mean	5.06	4.32	0.001	5.17	4.43	0.001
**Response latencies to trait judgments**						
Intelligence	5846	10384	0.000	5595	8737	0.024
Sociability	5484	8934	0.014	5213	8733	0.013
Athletic	5452	7936	0.011	5319	7885	0.010
Political Act.	5834	8387	0.007	5555	7358	0.105
Mean	5654	8910	0.000	5421	8178	0.002

A significant effect also occur between the trait assessed and the entitativity target *F*(1,116) = 12.890, *p* < 0.001; η_*p*_^2^ = 0.100. But, the exam of the means of this interaction show that only the scores of traits intelligence were not significantly different. Thus, with the exception of the traits intelligence, the participants in the high entitativity target reported significantly stronger traits ratings than the participants in the low entitativity target.

Neither the main effect of type of groups nor the interaction between entitativity and type of groups reached significance.

It was predicted that the level of perceived group entitativity would influence the way participants processed information about the group, more specifically, subjects were expected to make more on-line trait judgments about high rather than low entitativity groups, whether photo or text condition. The trait judgment response latencies were analyzed using a 2 (entitativity: high vs. low) × 2 (type of group presentation: photographs vs. description) × 4 (sociability, intelligence, political activism, athleticism Theme), ANOVA with repeated measures on the last variable.

The results support the prediction, the main effect of the entitativity target was significant *F*(1,116) = 33.394, *p* < 0.001; η_*p*_^2^ = 0.224. High entitativity group members were judged more quickly than low entitativity ones for both photo and description condition. No significant effects were found between the trait assessed and the other variables. Neither the main effect of type of groups nor the interaction between entitativity and type of groups reached significance.

#### Confidence in Judgment

Confidence in judgment was analyzed for each of the four trait judgments and in relation to the response latencies. This was analyzed with an ANOVA in 2 (entitativity: high vs. low) × 2 (type of group presentation: photographs vs. description) × 4 (sociability, intelligence, political activism, athleticism Theme), ANOVA with repeated measures on the last variable. The results support the prediction, the main effect of the entitativity target was significant *F*(1,116) = 10.358, *p* < 0.002; η_*p*_^2^ = 0.082. High entitativity group members were judged with more confidence than low entitativity ones for both photographs and description condition. A significant effect also occur between the trait assessed and the entitativity target *F*(1,116) = 10.396, *p* < 0.001; η_*p*_^2^ = 0.082. However the exam of the means of this interaction showed that it reflected variation in the magnitude of the differences in traits ratings among target but the pattern of those differences were the expected. Thus participants in the high entitativity target reported significantly stronger traits ratings (*M* = 4.54) than the participants in the low entitativity target (*M* = 3.90).

Neither the main effect of type of groups nor the interaction between entitativity and type of groups reached significance.

#### Transference of Group Characteristics to Individual Group Members

The last hypothesis predicted that perception of a group member would be affected by the degree of perceived entitativity. Brewer and Harasty (1996) proposed that perceivers have a prototypical representation of high entitativity groups and an exemplary representation of low entitativity groups. In other words, perceivers are more likely to treat high entitativity groups as collections and are therefore more likely to transfer group characteristics to their members. For low entitativity groups, perceivers are more likely to see the subject as an individual and are less likely to transfer the characteristics of the group to the individual.

Therefore, the previous prediction was that participants in the high entitativity group would be subject to more transference of behavioral traits from the group stereotype to individual group members, than participants in the low entitativity group.

**FIGURE 1:**
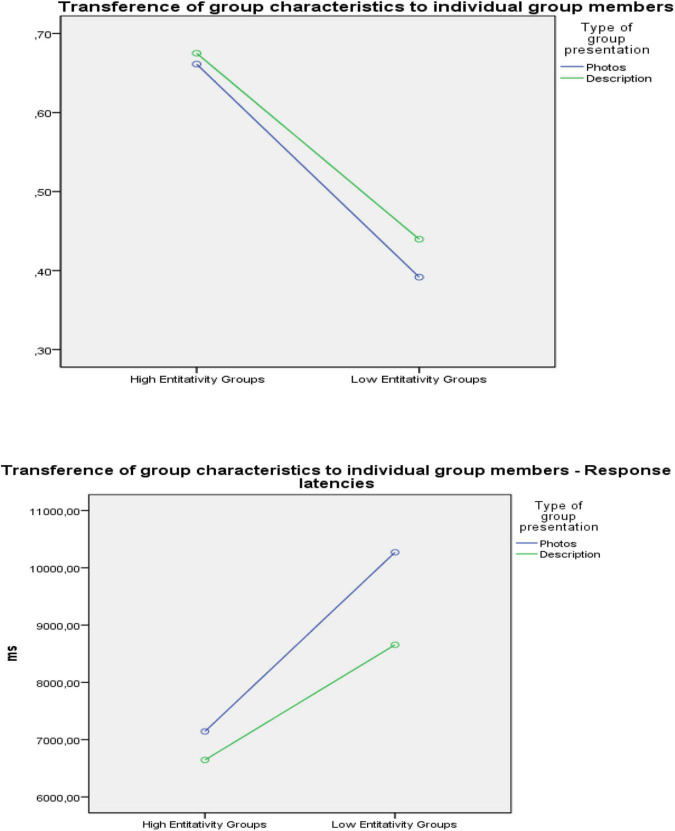
Transference of group characteristics to individual group members.

The percentage of traits correctly identified by each participant was collected in two different scores: inference scores and transference scores. The data was submitted to a ANOVA with 2 (entitativity: high vs. low) × 2 (type of group presentation: photographs vs. description) for each scores. The results showed a significant main effect on the transference scores *F*(1,116) = 30.563, *p* < 0.001; η_*p*_^2^ = 0.209. Participants on the high entitativity condition reported a higher transference scores (*M* = 0.668), than the participants on the low entitativity condition (*M* = 0.416). Neither the main effect of type of groups nor the interaction between entitativity and type of groups reached significance (see Figure 1).

The final analysis dealt with response latencies. The results showed a significant main effect on the transference response latencies *F*(1,116) = 24.668, *p* < 0.001; η_*p*_^2^ = 0.175. Participants in the transference test, the high entitativity group revealed significantly lower response latencies than the low entitativity group, in both the photo and description conditions (see Figure 1).

### Discussion

The study had two main aims. Firstly, it sought to replicate Study 2, using photographs or descriptions of neighborhoods, without any identification of the neighborhood’s name or location. The second objective was to evaluate the effect of perceived neighborhood entitativity on the transference of group stereotype to individual members.

The results regarding the first question supported the general hypothesis that the perception of entitativity on group impression formation for the photo condition. Thus, for higher entitativity groups, perceivers judged more extreme traits, made faster judgments and were more confident in rating sociability traits. Several previous studies confirm these results regarding groups with different levels of entitativity using different paradigms (e.g., Sanbonmatsu et al., 1987; McConnell et al., 1994; Susskind et al., 1999).

However, this study added significant information to the study of place of residence because it showed that people might evaluate the entitativity of a geographical space using only pictures of the neighborhood as information. This study confirmed the idea, presented before in Studies 1 and 2, that the physical and social characteristics of neighborhoods are interconnected and that both contribute to people’s perception of place entitativity. Thus, as physical appearance makes an important contribution to individuals’ impressions, it also is instrumental in the process of forming an impression of its residents.

On the contrary, individuals presented with descriptions rated the two groups differently, however, the difference was not significant. One possible explanation is that this specific description was not as effective as the photo in developing a clear perception of entitativity from the neighborhood.

This study also investigated how information about individual members is processed and transferred from one member to another. The results showed that perceivers made trait inferences about high and low entitativity groups, but more readily transferred these traits to other group members in high entitativity groups. In this sense, members of high entitativity groups are seen as interchangeable parts of the set, perceived as sharing the same attributes. This information has important implications for members of high entitativity groups, because they are perceived as being associated with the group stereotype more than with their individual characteristics. The results also revealed that high entitativity groups report significantly lower response latencies than low entitativity groups, in both the photo and description conditions. This means that, in the high entitativity condition, participants made an on-line abstraction of trait, leading to faster responses.

In highly entitative groups, upon the formation of first impressions, information about specific members is forgotten. To the extent that the group is treated as a cohesive and entitative unit, its members share all the attributes (Crawford et al., 2002). Thus, our findings support those of Crawford et al. (2002) who found a connection between entitativity and the way information is organized in forming impressions: category-based or person-based (Brewer, 1988; Fiske and Neuberg, 1990). In highly entitative groups, impressions are formed by integrating information about members into a general group impression. The impression of individual members is based on the group impression. In low entitativity groups, on the contrary, it is more difficult to develop an integrative impression of the group, and the impression of individual members is based more on the individual information retained, and less interchangeability of information occurs. Or, in consonance with Brewer et al. (2004), residents from highly entitative neighborhoods were more easily associated with prototypical representations while lesser entitative neighborhood members were represented by more exemplar-based processes.

These findings supported the idea of a relationship between entitativity and stereotypes (Brewer and Harasty, 1996) and supports Spencer-Rogers et al. ’s findings (2007) that entitativity predicts stereotyping for social categories and task groups.

## General Discussion

**FIGURE 2:**
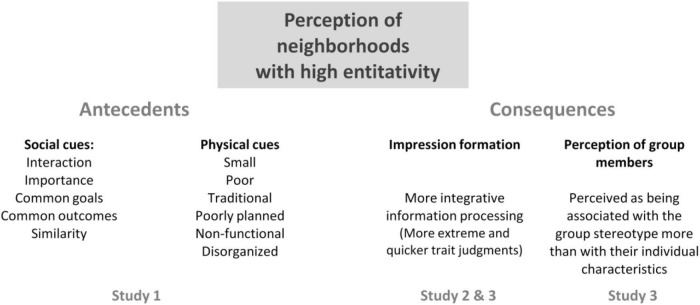
Results summary.

Firstly, these results confirm the relevance of using the concept of entitativity in understanding perceptions of membership in a geographical unit - neighborhood - as a subcategory of an urban area or city. Secondly, they confirm that group entitativity is important both when appealing to social perception of the neighborhood by way of name (Study 2) and when by physical characteristics (Study 3). Figure 2 presents a summary of the main results of the three studies.

The first study shows that the entitativity perception in the neighborhoods is highly correlated with some social group characteristics, as was found in other studies (described in the literature as antecedents of entitativity), but it is also highly correlated with some physical characteristics of the neighborhoods. It seems that some environmental cues about neighborhoods are sufficient to develop an impression about their entity. The perception of neighborhood entitativity has a set of cognitive consequences in processing information about the neighborhood and thinking about its residents. Furthermore, it seems that the photographs (of unknown places) are more efficient at eliciting entitativity processual consequences, which seems to suggest that group evaluation is a consistent process.

These findings are very interesting in the sense that this study confirms Lee’s assumption (1968, 2003) that a neighborhood is a knowledge structure that includes interdependent social and physical components, but has the same impact in terms of cognitive processes as other knowledge structures.

Studies 2 and 3 added significant information to the study of place of residence as a source of information for forming impressions. In fact, it confirmed that people can evaluate the entitativity of a geographical space, using only pictures of the neighborhood without any human presence as information, and that people make consistent inferences between the physical and social characteristics of the space, which have an impact on how they process information about the residents of that space. Information about the importance and accuracy of physical appearance, particularly in the formation of first impressions (e.g., Hassin and Trope, 2000; Zebrowitz and Montepare, 2008) was already available but places had previously been neglected in understanding the formation of impressions.

Social psychology traditionally has not taken into account the importance of physical environments in the formation of impressions. But intuitively we know that the spaces we build and the items we choose, give the observer many clues to our personalities, behaviors and values. As noted by James (1890), people use their home and neighborhoods as symbols of their personal prestige. Thus, places that people choose to live in are important sources of information and observers can learn a lot about us from those environments.

These studies offer a first look at the relationship between concept of entitativity and perception of urban spaces, and provide important information about the implication of urban design in impression formation. Perceived neighborhood entitativity has a set of effects on both information processing and the outcomes of that processing. Information in highly entitative neighborhoods is processed in an integrative way, which results, for example, in perception of group members as having the characteristics of the group, as we found in Study 3.

The conclusion of Study 3, that in highly entitative neighborhoods, the transference of social perception from the group to its members is easier, has important implications in terms of intergroup relationships in the urban context. In fact, neighborhood entitativity influences how information is processed as well as how individual residents are perceived. Therefore, place of residence can influence the strength or confidence in the use of the group stereotype in relation to its individual residents. This question must be explored further in future studies, in particular in a natural context.

From a theoretical point of view, this work clearly strengthens the argument that places can be seen as psychosocial features contributing to social identities, stereotypes, discrimination and self and alter judgments of groupness (entitativity) that shape social perception and behavior. In addition, the force of said phenomena can vary according to salience (Bernardo and Palma-Oliveira, 2013) and to the value that the group and individuals attribute to that belonging (ingroup) or to that perception (outgroup).

## Limitations and Future Directions

The studies herein are not free of limitations. Firstly, they are based on real categories (Lisbon neighborhoods), in the case of Studies 1 and 2, which prevents complete control of the variables that are the basis for subject classifications. On the other hand, Study 3 used photographs and descriptions, which despite being selected using predefined criteria, were not free of a certain degree of subjectivity. This may have resulted in presenting photographs of neighborhoods perceived as more entitative with a more sociopetal configuration of public space, i.e., which may lead to social interaction. Alternatively, in less entitative neighborhoods, photographs of more social-fugal spaces were selected. In this way we may have induced bias between physical and social characteristics, insofar as physical configurations may appeal to dimensions of social interaction.

Those physical cues related to the perception of a neighborhood as an entity (Study 1) must be thoroughly investigated. Future studies should, therefore, undertake photo manipulation in a laboratory environment to identify which physical characteristics and/or spatial configurations are associated with perceived entitativity.

Finally, another limitation of this study was the use of only male names in Study 3. Future studies should replicate the study with female names as well.

## Data Availability Statement

The data presented in this study are available on request. If there are relevant research needs, the data can be obtained by sending an email to FB. Please indicate the purpose of the research and the statement of data confidentiality in the email.

## Ethics Statement

The studies involving human participants were reviewed and approved by University of Évora. The patients/participants provided their written informed consent to participate in this study.

## Author Contributions

FB: conceptualization, methodology, formal analysis, and writing – original draft. JP-O: supervision and conceptualization. Both authors contributed to the article and approved the submitted version.

## Conflict of Interest

The authors declare that the research was conducted in the absence of any commercial or financial relationships that could be construed as a potential conflict of interest.

## Publisher’s Note

All claims expressed in this article are solely those of the authors and do not necessarily represent those of their affiliated organizations, or those of the publisher, the editors and the reviewers. Any product that may be evaluated in this article, or claim that may be made by its manufacturer, is not guaranteed or endorsed by the publisher.

## References

[B1] Abrams D. (2006). “The social psychology of neighbourliness,” in *Neighbourliness*, ed. Pilch T. (London: The Smith Institute), 24–36.

[B2] Bernardo F. Palma-Oliveira J. M. (2013). Place identity and place scale: the impact of place salience. *Psyecology* 4 167–193. 10.1007/s10897-011-9391-8 21826579 PMC3506010

[B3] Bernardo F. Palma-Oliveira J. M. (2016a). Identity to the neighborhood: discrimination and neighborhood size. *Self Identity* 15 579–598. 10.1080/15298868.2016.1178665

[B4] Bernardo F. Palma-Oliveira J. M. (2016b). Urban neighborhoods and intergroup relations: the importance of place identity. *J. Environ. Psychol.* 45 239–251. 10.1016/j.jenvp.2016.01.010

[B5] Bonaiuto M. Bilotta E. Bonnes M. Ceccarelli M. Martorella H. Carrus G. (2008). Local identity and the role of individual differences in the use of natural resources: the case of water consumption. *J. Appl. Soc. Psychol.* 38 947–967. 10.1111/j.1559-1816.2008.00333.x

[B6] Bonaiuto M. Breakwell G. M. Cano I. (1996). Identity processes and environmental threat: the effects of nationalism and local identity upon perception of beach pollution. *J. Commun. Appl. Soc. Psychol.* 6 157–175. 10.1002/(SICI)1099-1298(199608)6:3<157::AID-CASP367>3.0.CO;2-W

[B7] Brewer M. Harasty A. S. (1996). “Seeing groups as entities: the role of perceiver motivation,” in *Handbook of Motivation and Cognition: The Interpersonal Context*, Vol. 3 eds Sorrentino R. Higgings E. T. (New York, NY: Guildford Press), 347–370.

[B8] Brewer M. Weber J. Carini B. (1995). Person memory in intergroup contexts: categorization versus individuation. *J. Pers. Soc. Psychol.* 69 29–40. 10.1037/0022-3514.69.1.29

[B9] Brewer M. B. (1988). “A dual process model of impression formation,” in *Advances in Social Cognition*, Vol. 1 eds Srull T. Wyer R. (Hillsdale, NJ: Lawrence Earlbaum Associates).

[B10] Brewer M. B. Hong Y.-Y. Li Q. (2004). “Dynamic entitativity: perceiving groups as actors,” in *The Psychology of Group Perception: Perceived Variability, Entitativity, and Essentialism*, eds Yzerbyt V. Judd C. M. Corneille O. (New York, NY: Psychology Press), 25–38.

[B11] Brown B. Perkins D. D. Brown G. (2003). Place attachment in a revitalizing neighborhood: individual and block levels of analysis. *J. Environ. Psychol.* 23 259–271. 10.1016/s0272-4944(02)00117-2

[B12] Campbell D. T. (1958). Common fate, similarity, and other indices of the status of aggregates of persons as social entities. *Behav. Sci.* 3 24–25. 10.1002/bs.3830030103

[B13] Carlston D. E. (1994). “Associated system theory: a systematic approach to cognitive representations of persons,” in *Advances in Social Cognitions*, Vol. 7 eds Srull T. K. Wyer R. S. (Hillsdale, NJ: Lawrence Erlbaum Associates), 1–78. 10.1186/s12868-016-0283-6

[B14] Castano E. Sacchi S. Gries P. H. (2003). The perception of the other in international relations: evidence for the polarizing effect of entitativity. *Polit. Psychol.* 24 449–468. 10.1111/0162-895x.00336

[B15] Castano E. Yzerbyt V. Y. Paladino M.-P. Sacchi S. (2002). I belong, therefore I exist: ingroup identification, ingroup entitativity, and intergroup bias. *Pers. Soc. Psychol. Bull.* 28 135–143. 10.1177/0146167202282001

[B16] Crawford M. T. Sherman S. J. Hamilton D. L. (2002). Perceived entitativity, stereotype formation, and the interchangeability of group members. *J. Pers. Soc. Psychol.* 83 1076–1094. 10.1037//0022-3514.83.5.1076 12416913

[B17] Dasgupta N. Banaji M. R. Abelson R. P. (1999). Group entitativity and group perception: associations between physical features and psychological judgment. *J. Pers. Soc. Psychol.* 77 991–1003. 10.1037//0022-3514.77.5.991 10573876

[B18] Demoulin S. Leyens J. P. Yzerbyt V. (2006). Lay theories of essentialism. *Group Process. Intergroup Relat.* 9 25–42. 10.1177/1368430206059856

[B19] Félonneau M. L. (2004). Love and loathing of the city: urbanophilia and urbanophobia, topological identity and perceived incivilities. *J. Environ. Psychol.* 24 43–52. 10.1016/s0272-4944(03)00049-5

[B20] Fiske S. T. Neuberg S. L. (1990). “A continuum of impression formation, from category-based to individuating processes: influences of information and motivation on attention and interpretation,” in *Advances in Experimental Social Psychology*, Vol. 23 ed. Zanna M. P. (New York, NY: Academic Press), 1–74. 10.1016/S0065-2601(08)60317-2

[B21] Gaertner L. Schopler J. (1998). Perceived ingroup entitativity and intergroup bias: an interconnection of self and others. *Eur. J. Soc. Psychol.* 28 963–980. 10.1002/(sici)1099-0992(1998110)28:6<963::aid-ejsp905>3.0.co;2-s

[B22] Galster G. (2001). On the nature of neighborhood. *Urban Stud.* 38 2111–2124. 10.1080/00420980120087072

[B23] Gosling S. D. Gaddis S. Vazire S. (2008). “First impressions based on the environments we create and inhabit,” in *First Impressions*, eds Ambady N. Skowronski J. J. (New York, NY: Guilford), 334–356.

[B24] Gosling S. D. Ko S. J. Mannarelli T. Morris M. E. (2002). A room with a cue: judgments of personality based on offices and bedrooms. *J. Pers. Soc. Psychol.* 82 379–398. 10.1037/0022-3514.82.3.379 11902623

[B25] Hamilton D. L. Sherman S. J. (1996). Perceiving persons and groups. *Psychol. Rev.* 2 336–355. 10.1037/0033-295X.103.2.336 8637962

[B26] Hamilton D. L. Sherman S. J. Castelli L. (2002). “A group by any other name – the role of entitativity in group perception,” in *European Review of Social Psychology*, Vol. 12 eds Stroebe W. Hewstone M. (Hoboken, NJ: John Wiley & Sons Ltd), 139–166. 10.1080/14792772143000049

[B27] Hamilton D. L. Sherman S. J. Lickel B. (1998). “Perceiving social groups: the importance of entitativity continuum,” in *Intergroup Cognition and Intergroup Behavior*, eds Sedikides C. Schopler J. Insko C. A. (Mahwah, NJ: Erlbaum), 47–74.

[B28] Hamilton D. L. Sherman S. J. Maddox K. B. (1999). “Dualities and continua: implications for understanding perceptions of persons and groups,” in *Dual-Process Theories in Social Psychology*, eds Chaiken S. Trope Y. (New York, NY: Guilford), 606–626.

[B29] Haslam N. Rothschild L. Ernst D. (2004). “Essentialism and entitativity: structures of beliefs about the ontology of social categories,” in *The Psychology of Group Perception. Perceived Variability, Entitativity, and Essentialism*, eds Yzerbyt V. Judd C. M. Corneille O. (New York, NY: Psychology Press), 61–78. 10.4324/9780203644973

[B30] Hassin R. Trope Y. (2000). Facing faces: studies on the cognitive aspects of physiognomy. *J. Pers. Soc. Psychol.* 19 115–122. 10.1037//0022-3514.78.5.837 10821193

[B31] Hogg M. A. Sherman D. K. Dierselhuis J. Maitner A. T. Moffitt G. (2007). Uncertainty, entitativity, and group identification. *J. Exp. Soc. Psychol.* 43 135–142. 10.1016/j.jesp.2005.12.008

[B32] James W. (1890). *The Principles of Psychology.* New York, NY: Holt. 10.1037/10538-000

[B33] Johnson A. L. Queller S. (2003). The mental representations of high and low entitativity groups. *Soc. Cogn.* 21 101–119. 10.1521/soco.21.2.101.21316

[B34] Kasmar J. (1970). The development of a usable lexicon of environmental descriptors. *Environ. Behav.* 2 153–164. 10.1177/001391657000200202

[B35] Lee T. (1968). Urban neighborhood as a socio-spatial scheme. *Hum. Relat.* 21 241–267. 10.1177/001872676802100303

[B36] Lee T. (1976). *Psychology and the Environment.* London: Methuen.

[B37] Lee T. (2003). “Schema theory and the role of socio-spatial schemata in environmental psychology,” in *Psychological Theories for Environmental Issues*, eds Bonnes M. Lee T. Bonaiuto M. (Aldershot: Ashgate), 27–62.

[B38] Lewicka M. Rowiński K. Iwańczak B. Bałaj B. Kula A. M. Oleksy T. (2019). On the essentialism of places: between conservative and progressive meanings. *J. Environ. Psychol.* 65:101318. 10.1016/j.jenvp.2019.101318

[B39] Lewis A. C. Sherman S. J. (2010). Perceived entitativity and the black-sheep effect: when will we denigrate negative ingroup members? *J. Soc. Psychol.* 150 211–225. 10.1080/00224540903366388 20397595

[B40] Lickel B. Hamilton D. L. Wieczorkowska G. Lewis A. Sherman S. J. Uhles A. N. (2000). Varieties of groups and the perception of group entitativity. *J. Pers. Soc. Psychol.* 78 223–246. 10.1037/0022-3514.78.2.223 10707331

[B41] Lynch K. (1976). *Managing a Sense of Region.* Cambridge, MA: MIT Press.

[B42] Maroco J. (2021). *Análise Estatística.* Lisboa: Edições Sílabo.

[B43] McConnell A. Sherman S. J. Hamilton D. L. (1997). Target entitativity: implications for information processing about individual and group targets. *J. Pers. Soc. Psychol.* 72 750–762. 10.1037/0022-3514.72.4.750 9108693

[B44] McConnell A. R. Sherman S. J. Hamilton D. L. (1994). On-line and memory based aspects of individual and group target judgments. *J. Pers. Soc. Psychol.* 67 173–185. 10.1037/0022-3514.67.2.173 7932060

[B45] McGarty C. Haslam S. A. Hutchinson K. J. Grace D. M. (1995). Determinants of perceived consistency: the relationship between group entitativity and the meaningfulness of categories. *Br. J. Soc. Psychol.* 34 237–256. 10.1111/j.2044-8309.1995.tb01061.x 7551771

[B46] Mullen B. (1991). Group composition, salience, and cognitive representations: the phenomenology of being in group. *J. Exp. Soc. Psychol.* 27 297–323. 10.1016/0022-1031(91)90028-5

[B47] Norberg-Schultz C. (1980). *Genius Loci: Towards a Phenomenology of Architecture.* New York, NY: Rizolli.

[B48] Perez-Lopez R. Aragones J. I. Amerigo M. (2017). Primary spaces and their cues as facilitators of personal and social inferences. *J. Environ. Psychol.* 53 157–167. 10.1016/j.jenvp.2017.07.008

[B49] Pickett C. L. (2001). The effects of entitativity beliefs on implicit comparisons between group members. *Pers. Soc. Psychol. Bull.* 27 515–525. 10.1177/0146167201275001

[B50] Pickett C. L. Perrott D. A. (2004). Shall I compare thee? Perceived entitativity and ease of comparison. *J. Exp. Soc. Psychol.* 40 283–289. 10.1016/S0022-1031(03)00121-5

[B51] Poggio L. Aragonés J. I. Pérez-López R. (2013). Inferences of personality traits from bedroom objects: an approach from the SCM. *Procedia Soc. Behav. Sci.* 82 668–673. 10.1016/j.sbspro.2013.06.327

[B52] Pol E. Moreno E. Guàrdia J. Iniguez L. (2002). Identity, quality of life, and sustainability in an urban suburb of Barcelona. Adjustment to the city-identity-sustainability network structural model. *Environ. Behav.* 34 67–80. 10.1177/0013916502034001005

[B53] Rogier A. Yzerbyt V. Y. (1999). Social attribution: the role of homogeneity in subjective essentialism. *Swiss J. Psychol.* 54 233–240.

[B54] Ross L. Amabile T. M. Steinmetz J. L. (1977). Social roles, social control and biases in social perception processes. *J. Pers. Soc. Psychol.* 35 485–494. 10.1037/0022-3514.35.7.485

[B55] Sanbonmatsu D. M. Sherman S. J. Hamilton D. L. (1987). Illusory correlation in the perception of individuals and groups. *Soc. Cogn.* 5 1–25. 10.1521/soco.1987.5.1.1

[B56] Slovic P. Fischhoff B. Lichtenstein S. (1985). “Characterizing perceived risk,” in *Perilous Progress: Managing the Hazards of Technology*, eds Kates R. W. Hohenemser C. Kasperson J. X. (Boulder, CO: Westview), 91–125.

[B57] Spencer-Rodgers J. Hamilton D. L. Sherman S. J. (2007). The central role of entitativity in stereotypes of social categories and task groups. *J. Pers. Soc. Psychol.* 92 369–388. 10.1037/0022-3514.92.3.369 17352598

[B58] Stedman R. C. (2002). Towards a social psychology of place: predicting behaviour from place-based cognitions, attitude and identity. *Environ. Behav.* 34 561–581. 10.1177/0013916502034005001

[B59] Susskind J. Maurer K. Thakkar V. Hamilton D. L. Sherman J. W. (1999). Perceiving individuals and groups: expectancies, dispositional inferences, and causal attributions. *J. Pers. Soc. Psychol.* 76 181–191. 10.1037/0022-3514.76.2.181 10074704

[B60] Tajfel H. Turner J. C. (1979). “An integrative theory of intergroup conflict,” in *The Social Psychology of Intergroup Relations*, eds Austin W. G. Worchel S. (Monterey, CA: Brooks/Cole), 7–24.

[B61] Thakkar V. (2001). *The Role of Entitativity in Judgments About Groups.* Doctoral dissertation. Santa Barbara, CA: University of California.

[B62] Uzzell D. Pol E. Badenas D. (2002). Place identification, social cohesion, and environmental sustainability. *Environ. Behav.* 34 26–53. 10.1177/0013916502034001003

[B63] Valera S. Guardia J. (2002). Urban social identity and sustainability Barcelona’s Olympic village. *Environ. Behav.* 34 54–66. 10.1177/0013916502034001004

[B64] Vaske J. J. Kobrin K. C. (2001). Place attachment and environmentally responsible behaviour. *J. Environ. Educ.* 32 16–21. 10.1080/00958960109598658

[B65] Vorkinn M. Riese H. (2001). Environmental concern in a local context. The significance of place attachment. *Environ. Behav.* 33 249–263. 10.1177/00139160121972972

[B66] Wells M. Thelen L. (2002). What does your workspace say about you? *Environ. Behav.* 34 300–321. 10.1177/0013916502034003002

[B67] Wells M. M. Thelen L. Ruark J. (2007). Workspace personalization and organizational culture. *Environ. Behav.* 39 616–634. 10.1177/0013916506295602

[B68] Wnuk A. Oleksy T. Toruńczyk-Ruiz S. Lewicka M. (2021). The way we perceive a place implies who can live there: essentialisation of place and attitudes towards diversity. *J. Environ. Psychol.* 75:101600. 10.1016/j.jenvp.2021.101600

[B69] Yzerbyt V. Y. Castano E. Leyens J.-P. Paladino M.-P. (2000). The primacy of the ingroup: the interplay of entitativity and identification. *Eur. Rev. Soc. Psychol.* 11 257–295. 10.1080/14792772043000059

[B70] Yzerbyt V. Y. Rogier A. Fiske S. T. (1998). Group entitativity and social attribution: on translating situational constraints into stereotypes. *Pers. Soc. Psychol. Bull.* 24 1089–1103. 10.1177/01461672982410006

[B71] Zebrowitz L. A. Montepare J. M. (2008). Social psychological face perception: why appearance matters. *Soc. Pers. Psychol. Compass* 2 1497–1517. 10.1111/j.1751-9004.2008.00109.x 20107613 PMC2811283

