# Corrigendum: Tell me where you live… How the perceived entitativity of neighborhoods determines the formation of impressions about their residents

**DOI:** 10.3389/fpsyg.2023.1201521

**Published:** 2023-04-28

**Authors:** Fátima Bernardo, José Manuel Palma-Oliveira

**Affiliations:** ^1^Department of Psychology, University of Évora, Évora, Portugal; ^2^CITUA - Center for Innovation in Territory, Urbanism, and Architecture, IST, Universidade de Lisboa, Lisbon, Portugal; ^3^CICPSI, Research Center for Psychological Science, Faculdade de Psicologia, Universidade de Lisboa, Lisbon, Portugal

**Keywords:** entitativity, impression formation, neighborhood, intergroup relationships, stereotypes

In the published article, the Funding statement was omitted in error. The correct Funding statement appears below.

